# Altering product placement to create a healthier layout in supermarkets: Outcomes on store sales, customer purchasing, and diet in a prospective matched controlled cluster study

**DOI:** 10.1371/journal.pmed.1003729

**Published:** 2021-09-07

**Authors:** Christina Vogel, Sarah Crozier, Daniel Penn-Newman, Kylie Ball, Graham Moon, Joanne Lord, Cyrus Cooper, Janis Baird

**Affiliations:** 1 Medical Research Council Lifecourse Epidemiology Unit, University of Southampton, Southampton General Hospital, Southampton, United Kingdom; 2 NIHR Southampton Biomedical Research Centre, University Hospital Southampton NHS Foundation Trust and University of Southampton, Southampton, United Kingdom; 3 Institute for Physical Activity and Nutrition Research, School of Exercise and Nutrition Sciences, Deakin University, Geelong, Australia; 4 School of Geography and Environmental Science, University of Southampton, Southampton, United Kingdom; 5 Southampton Health Technology Assessments Centre, Wessex Institute, University of Southampton, Southampton, United Kingdom; University of Cambridge, UNITED KINGDOM

## Abstract

**Background:**

Previous product placement trials in supermarkets are limited in scope and outcome data collected. This study assessed the effects on store-level sales, household-level purchasing, and dietary behaviours of a healthier supermarket layout.

**Methods and findings:**

This is a prospective matched controlled cluster trial with 2 intervention components: (i) new fresh fruit and vegetable sections near store entrances (replacing smaller displays at the back) and frozen vegetables repositioned to the entrance aisle, plus (ii) the removal of confectionery from checkouts and aisle ends opposite. In this pilot study, the intervention was implemented for 6 months in 3 discount supermarkets in England. Three control stores were matched on store sales and customer profiles and neighbourhood deprivation. Women customers aged 18 to 45 years, with loyalty cards, were assigned to the intervention (*n* = 62) or control group (*n* = 88) of their primary store. The trial registration number is NCT03518151. Interrupted time series analysis showed that increases in store-level sales of fruits and vegetables were greater in intervention stores than predicted at 3 (1.71 standard deviations (SDs) (95% CI 0.45, 2.96), *P* = 0.01) and 6 months follow-up (2.42 SDs (0.22, 4.62), *P* = 0.03), equivalent to approximately 6,170 and approximately 9,820 extra portions per store, per week, respectively. The proportion of purchasing fruits and vegetables per week rose among intervention participants at 3 and 6 months compared to control participants (0.2% versus −3.0%, *P* = 0.22; 1.7% versus −3.5%, *P* = 0.05, respectively). Store sales of confectionery were lower in intervention stores than predicted at 3 (−1.05 SDs (−1.98, −0.12), *P* = 0.03) and 6 months (−1.37 SDs (−2.95, 0.22), *P* = 0.09), equivalent to approximately 1,359 and approximately 1,575 fewer portions per store, per week, respectively; no differences were observed for confectionery purchasing. Changes in dietary variables were predominantly in the expected direction for health benefit. Intervention implementation was not within control of the research team, and stores could not be randomised. It is a pilot study, and, therefore, not powered to detect an effect.

**Conclusions:**

Healthier supermarket layouts can improve the nutrition profile of store sales and likely improve household purchasing and dietary quality. Placing fruits and vegetables near store entrances should be considered alongside policies to limit prominent placement of unhealthy foods.

**Trial registration:**

ClinicalTrials.gov NCT03518151 (pre-results)

## Abstract 2

Christina Vogel and colleagues assess the effects of creating a healthier layout in supermarkets in England on store-level sales, household-level purchasing, and dietary behaviors of women customers.

## Author summary

**Why was this study done?:**

- Supermarkets are a major source of food for many families, and in-store marketing techniques like prominent product placement can influence customers’ food choices.
- Studies testing whether placement strategies can promote healthier food purchasing have been limited in scope, placing healthy products alongside unhealthy products or a single location (i.e., checkouts), and findings have been inconsistent.
- Governments are proposing to ban the use of prominent placement strategies for unhealthy food and drink products in supermarkets, yet evidence from robustly designed real-world studies remains limited.

**What did the researchers do and find?:**

- We assessed whether creating a healthier layout in discount supermarkets in England improves the healthiness of foods bought by all customers who visit the stores, as well as foods purchased and eaten by women customers aged 18 to 45 years.
- We found that storewide confectionery sales declined, and fruit and vegetable sales increased, when nonfood items and water were placed at checkouts and aisle ends opposite, and an expanded fruit and vegetable section was repositioned near the store entrance; women’s fruit and vegetable purchasing and dietary quality improved, but their confectionery purchasing did not.

**What do these findings mean?:**

- Our results should be viewed with some caution; because of some limitations in the design of the study, the results may be over- or underestimated. However, bans on prominent placement of unhealthy foods in supermarkets could be beneficial for population diet, and effects may be even better if supermarkets were also required to place produce near their entrances.

## 1. Introduction

Obesity and poor diet constitute 2 of the greatest threats to the population health [1,2]. They are costly to society [3] and disproportionately affect those who are socioeconomically disadvantaged [4]. The importance of improving population diet is ever more apparent with obesity and poor diet emerging as key risk factors for a severe response to the Coronavirus Disease 2019 (COVID-19) infection, particularly among adults aged under 65 years [5,6]. Most families rely on supermarkets for their food [7]; during COVID-19 lockdown, this reliance increased [8]. Such dependence on supermarkets as a primary food source makes them an appropriate setting for interventions to improve dietary behaviours. Despite online grocery sales increasing from 7% to 10% of the United Kingdom market recently [9], the majority of sales occur in-store where customers are exposed to marketing techniques that attempt to influence their food choices and preferences [10,11]. Product placement is one marketing technique used in supermarkets that predominantly promotes unhealthy food and beverage choices. For example, in the UK, two-thirds of all food and drink products placed in prominent in-store locations, such as checkouts, store entrances, end of aisles, and freestanding display units, have been found to be sugary or calorie-dense, ultraprocessed products; less than 1% of food products positioned in these prominent supermarket locations were fruits or vegetables [12]. This finding is a concern for population health, with growing evidence that these placement strategies can prompt customers to buy these unhealthy products [13].

In an effort to curb the influence of unhealthy marketing tactics on population diet, the UK government announced their intention to ban the use of prominent placement strategies for unhealthy food and drink products in supermarkets and other outlets [14]. This proposal forms part of the national strategy to address childhood obesity [15]. There is a pressing need for further evidence from local, well-designed intervention studies aimed at testing the effect, and cost impact, of healthier product placement strategies. Such evidence could assist UK policy makers appropriately frame the proposed ban, as well as help guide future government intervention to improve diet across the world.

Previous studies testing the effect of “healthier checkouts” in supermarkets, which placed healthier snack items alongside or at alternate checkouts to unhealthy snacks, have shown limited success at reducing sales of unhealthy food and beverages [16–18]. Few supermarket trials have tested the effects of removing unhealthy products from all checkouts, and none, to our knowledge, have additionally tested the effects of removing unhealthy products from all the end-of-aisle displays opposite checkouts. Furthermore, little prior research has considered the impact of replacing unhealthy food products at checkout areas with nonfood items in an effort to restrict opportunities for impulsive calorie purchases while aiming to preserve impulse expenditure. Positioning products near the store entrance is another prominent placement strategy used by supermarkets to tempt customers [19].

While many supermarkets do place fresh fruits and vegetables in a position that customers encounter when first entering the store, a number of discount and small supermarket chains do not routinely place fruits and vegetables near the store entrance. UK research shows that discount and small supermarkets have less healthy environments than other UK supermarkets, including lower availability and less prominent placement of fresh fruits and vegetables [20]. These poorer in-store environments may be contributing to dietary inequalities because families experiencing disadvantage and younger adults, known to have poorer quality diets, frequently rely on these stores for their food [21,22]. Retail intervention studies in the United States have shown promising effects on food purchasing habits among low-income, minority groups when fresh fruit and vegetable displays were moved to the front of the store [23,24]. Evaluation of the effects of such a strategy, in combination with the removal of confectionery from checkouts, is needed and could inform future government policy.

Healthier product placement interventions could have economic implications for supermarkets and individuals with potential impact on commercial viability and household food shopping budgets, respectively. Economic evaluations of health-related supermarket interventions have been rarely considered, yet such information is needed to inform future policy action [25].

This study will help to address current evidence gaps regarding the use of prominent placement strategies to support improvements in population diet. It aims to assess whether creating a healthier layout in discount supermarkets in England improves the healthiness of store sales (primary outcome) and the purchasing and dietary behaviours of women customers aged 18 to 45 years (secondary outcomes) after 3 and 6 months. To our knowledge, this study is unique in its analysis of individual loyalty card data, in addition to store sales data, as well as collecting dietary data from more than 1 family member [13,26]. The study also evaluated possible cost implications of the intervention from individual and retailer perspectives.

## 2. Methodology

### 2.1 Study design and setting

This was a pilot study with a prospective matched controlled cluster design, with participants clustered within 6 study supermarkets to account for the store-based intervention. The flow diagram, Fig A in S1 File, illustrates the time frame of store sales, participant purchasing dietary data collection. The study, which took place between April 2016 and March 2017, was approved by the University of Southampton, Faculty of Medicine Ethics Committee (ID 20986.A2) and was conducted in accordance with the Declaration of Helsinki and data protection regulations; it was registered with ClinicalTrials.gov (NCT03518151, pre-results).

The setting for this study was stores of a discount supermarket chain located in more socioeconomically deprived neighbourhoods (within the most deprived 5 Index of Multiple Deprivation (IMD) deciles [27]) across England. The collaborating supermarket has over 900 stores nationwide and holds approximately 2% of the grocery market share in the UK [28].

This pilot study sampled 6 stores, 3 intervention and 3 control stores. The number of stores included was determined by the refurbishment schedule of the supermarket chain. Recruitment of additional stores and randomisation of stores were not viable within the company’s business model; intervention stores were selected because structural changes to their in-store environment had already been planned at the time of the study by the company, had an average sales profile, and were located in areas with higher neighbourhood deprivation. Control stores were matched to an intervention store based on (i) sales profile; (ii) customer profile; and (iii) neighbourhood deprivation (IMD). Matching on these factors aimed to increase the similarity of intervention and control stores and reduce the effects of confounding. Control stores were geographically distant from intervention stores to reduce contamination effects of control women shopping at intervention stores.

### 2.2 Intervention and control conditions

The intervention was implemented continuously for 6 months and had 2 components executed simultaneously: (i) more prominent placement of fruits and vegetables; and (ii) removal of unhealthy foods from checkouts and the end-of-aisle opposite checkouts. The first intervention component involved expanding the produce section to increase the availability of fresh fruits and vegetables and positioning the produce near the store entrance. Frozen vegetables were also relocated to the first aisle, a more prominent position in store. All unhealthy foods (confectionery, crisps, biscuits, etc.), but predominantly confectionery (chocolate, sweets, or candy), were removed from all checkouts and displays at the end-of-aisle opposite checkouts and replaced with nonfood items (i.e., tissues, painkillers, lip balm, cleansing wipes, toothpaste, soap, deodorant, and hand wash), water, and sugar-free gum. One intervention store also positioned some fresh fruits and vegetables at the checkouts because of the size and shape of the checkout display unit. In each intervention store, the confectionery section was moved to the least prominent position, the last aisle of the store. Seasonal confectionery (i.e., Easter, Christmas, Mother’s/Father’s Day, Valentine’s Day, and Halloween) and branded confectionery for which marketing space was already paid was positioned at the store entrance and in freestanding displays in the aisles but not at the checkouts or end-of-aisle opposite checkouts during the intervention period. Intervention stores also underwent improvements in presentation (e.g., cleaning, painting, and updated signage) at the time the intervention was implemented.

The control condition was the previous layout of stores, as at the baseline period, with (i) a limited range of fresh fruits and vegetables, placed at the back of the store; (ii) frozen vegetables placed in a middle aisle of the store; and (iii) confectionery placed at the checkouts and the confectionery section positioned at end-of-aisle opposite checkouts.

### 2.3 Participant eligibility and recruitment

Women of childbearing age were targeted for this intervention because of their role as household food gatekeepers [29] and their influence on the short-and long-term health of the next generation [30]. For the children of these women, establishing good dietary habits early in life is important for optimal growth, development, and long-term health [31]. Eligible participants were women, aged 18 to 45 years, who held a loyalty card with the study supermarket chain and had shopped in a study store in the 12 weeks before recruitment (according to loyalty card data). Women under the age of 18 or over 45 years at the time of the study who did not hold a loyalty card or only shopped online were not eligible to participate.

Recruitment occurred in 3 waves between July and September 2016. Women from each pair of stores were recruited over the same period, prior to the implementation period for that intervention store. Eligible women in all 6 study stores, identified from the loyalty card register, were sent a letter inviting them to participate in a study that was investigating the food shopping and eating patterns of women aged 18 to 45 years. The letter did not contain details about the intervention. The letter was sent by the supermarket on behalf of the research team in order to comply with data protection laws. Interested women contacted the research team directly via freephone number, text, or email and were screened for eligibility and then provided informed consent.

In addition to being mailed a letter, participants in the first pair of stores were initially contacted by the supermarket via email and text message, and advertisements about the study were placed on the back of shopping receipts and on Facebook. These additional recruitment methods, however, yielded very little interest from participants and were thus phased out over the duration of the study. In order to boost participant numbers in the first pair of stores, in-store recruitment was used, whereby members of the research team approached women customers while shopping and provided them with a study information sheet. Interested women registered with the researcher in-store and were subsequently phoned and consented. This method proved effective at enhancing representation of disadvantaged customers and was used for all 6 study stores. To promote retention, all participants were offered up to 3× £10 Love2Shop vouchers for taking part in the study. For comparison, the national minimum living wage rate for adults over 25 years in 2016 was £7.20/hour. Love2Shop vouchers are multioption vouchers that can be used at 150 leading high street retailers, which span a range of retail categories.

### 2.4 Outcome measures

The data collected included 9 months continuous store sale transaction data (primary outcome) and participant loyalty card data (secondary outcome) provided by the collaborating supermarket to cover 3 time periods: time (1) baseline (3 months prior to intervention implementation; time (2) short-term intervention effects (0 to 3 months postintervention commencement because evidence suggests that this represents an appropriate interval for habit formation [32]; and time (3) longer-term intervention effects (3 to 6 months postintervention commencement to assess sustained behavioural changes). In order to obtain an understanding of intervention effects on household members’ diets (secondary outcomes), interview-administered telephone questionnaires obtained information about participants’ diets and diets of their children aged 2 to 6 years (where applicable) at 3 time points: baseline (time 1) and 3 (time 2) and 6 months (time 3) following intervention commencement.

Store sales of fresh fruits and vegetables, frozen vegetables, confectionery, and intervention checkout items were provided as numbers of items for each product sold in each week of the study period. Participant purchasing data covering the same categories were provided as the number of items for each product purchased at each store visit during the study period. The research team aggregated these purchasing data from each visit to a weekly structure for analysis to enable our data to be presented as items per household per week in order to be able to detect changes in visit frequency as well as purchasing quantity, a method used in previous supermarket trials [33,34]. Store closure for structural changes to intervention stores affected 2 weeks of sales and purchasing data; these 2 weeks of data were removed from the analysis for both the intervention and matched control stores. Christmas notably impacted another 2 weeks, requiring a further 2 weeks of data to be removed. Store sales and individual purchasing datasets consisted of the 11 weeks prior to the intervention and 24 weeks afterward.

Measures of women’s dietary quality, and their young children’s dietary quality (where relevant), were assessed using published, validated tools [35,36]. Participants were asked to indicate how often in the previous month they (or their child) consumed each of 20 foods in a Food Frequency Questionnaire (FFQ). A dietary quality score for each woman (or child) was calculated by multiplying their reported frequency of consumption of each of the 20 items from their FFQ by corresponding weightings derived from the published tools (based on principal component analysis) and then summing the results. Dietary quality scores were then standardised to have a mean of 0 and standard deviation (SD) of 1. Higher scores represent better dietary quality characterised by higher intakes of vegetables, fruit, water, and whole grain bread and lower intakes of white bread, processed meats, chips, crisps, and sugar. Women’s daily fruit and vegetable intake was measured using a 2-item tool [37]. This measure details the amount (quantity) of fruits and vegetables eaten and complemented the frequency data collected by the FFQ.

The financial effects of the intervention on stores and women was assessed by calculating changes in total weekly store sales and changes in the amounts of money participants spent on grocery foods per week respectively, from before to after the intervention. Participants reported, at each survey wave, the total amount of money they spent on groceries in the past month. Participants’ weekly household spend on groceries at the collaborating supermarket chain were provided by the loyalty card data, and weekly total sales for each store were provided by the store transaction data.

### 2.5 Fidelity assessment

All stores were visited by a member of the research team during the baseline period prior to intervention implementation to assess whether the preintervention and control layouts were similar for each pair of stores. Post-intervention visits and phone calls were made to all stores to assess fidelity of both control and intervention conditions using photographic monitoring and discussions with supermarket staff.

### 2.6 Statistical analysis

Descriptive variables are given as percentage (frequency) for categorical variables and median (interquartile range) for nonnormally distributed continuous variables. Differences between intervention and control participants were tested using chi-squared tests for categorical variables and Mann–Whitney rank sum tests for nonnormally distributed continuous variables.

The distributions of the data were unknown in advance of study commencement because this was a pilot study. The research team therefore made decisions about the statistical analyses following (i) completion of initial analyses to determine the shape of the outcome data; and (ii) consultation with the literature to identify appropriate statistical methods for the various outcome variables.

Store sales data were analysed using an interrupted time series [38]. Weekly sales per store of fresh fruits and vegetables, frozen vegetables, confectionery, and intervention checkout items were transformed to normal distributions using inverse normal (also known as Fisher–Yates) transformations [39] to protect commercially sensitive sales figures. Time series models were fitted with terms for study week (linear term and weeks from baseline), intervention, level (an indicator of the postintervention period), trend (study week in the postintervention period), and interactions between intervention and study week, intervention and level, and intervention and trend. By including the variable “level,” the model will test for a step change at the time of the intervention because this immediate effect was anticipated based on conceptual models of consumer behaviour and evidence from existing supermarket placement studies [13,40]. The time series models were fitted separately in each pair of stores in order to account for the store pairing in the analyses. For the confectionery outcome, there was a strong pattern of steady increase in sales until Christmas, an abrupt fall in sales over the Christmas period, and a steady increase in sales after Christmas; therefore, an additional term for post-Christmas level was included in the confectionery models. The *P* value for the interaction between intervention and level indicates the significance of the impact of the intervention on level of store sales at the time of the intervention. A counterfactual line is included on the interrupted time series graphs, indicating the trends in sales that would have been expected had the intervention not occurred. Confidence intervals at 3 and 6 months postintervention were calculated using the delta method [41]. In order to inform planning of future cluster trials, we also fitted a random effects multilevel linear regression model in order to calculate an intraclass correlation coefficient (ICC). The outcome variable was baseline weekly sales per store of fresh fruit and vegetable z-score, and store ID was used to define clustering.

Fixed-effects meta-analysis [42] was used to synthesise the differences between pairs of stores at the time of intervention, 3 and 6 months postintervention. This method was selected to enable the (i) retention of the store pairing in the study design; (ii) comparisons between pairs; and (iii) overall statements of study effect size and precision [43]. Results were interpreted on the original items sold per week scale by calculating the equivalent change on the original scale to the change from the median on the Fisher–Yates transformed scale. The collaborating supermarket chain sells only packaged fruits and vegetables (products were not sold singly), with each item averaging 5 portions (approximately 400 g). Similarly, confectionery sales data indicated that the most popular items weighed 100 to 200 g. Thus, applying national recommendations [44] each item was considered equivalent to approximately 3 portions (150g); this portion size information was used to convert results from items to portions.

For the individual purchasing data, it was not possible to use a time series analysis because the data had a strong right-hand skew; for example, 83% of the women’s weekly purchases resulted in no sales of fresh fruits and vegetables. The outcome data were therefore dichotomised to indicate whether each week resulted in any purchases of the food category under consideration. A difference-in-difference approach was used, in line with previous supermarket placement research [17], so that each logistic regression model included fixed effects for intervention group, time period, and the interaction between intervention group and time period. Time period was coded as 2 dummy variables indicating the 0 to 3 and 3 to 6 months periods postintervention. The interaction terms test whether the difference between purchasing during the intervention compared to during the preintervention period differed between intervention and control stores. In addition, random effects were included for women, to account for the multilevel structure of the data, with weeks clustered within women; there was an insufficient number of stores in this study to include random effects for stores. Women’s data were analysed according to the store they were recruited from in order to conform to an intention-to-treat analysis.

The effects of the intervention on changes in diet from baseline to 3 and 6 months postintervention were explored using linear regression models with diet as the outcome and intervention group, diet at baseline, and IMD (to control for similarities between pairs of stores) as predictors. A second set of regression models included confounding variables determined prior to analysis using a directed acyclic graph (DAG) (Figs B and C in S1 File) [45]; variables included as confounding variables were age, money spent on groceries, number of children in the household, and woman’s education.

Changes in amounts of money spent on grocery foods per week were analysed using a difference-in-difference approach [17]. The distribution of the amounts of money spent on grocery foods was right-skewed, so a log transformation was applied. A linear regression model was fitted including effects for intervention group, time period, and the interaction between intervention group and time period. Time period was coded as 2 dummy variables indicating the 0-3 and 3-6 month periods postintervention. The interaction terms test whether the difference between the amounts of money spent on grocery foods during the intervention compared to during the preintervention period differed between intervention and control stores. Total individual purchasing data were similarly analysed using a difference-in-difference approach based on a linear regression model with average weekly purchases in pounds (£) as the outcome and interaction terms (as described for the reported money spent on grocery foods). Total stores sales data were analysed using an interrupted time series in the same way as the confectionery data (i.e., an additional term for post-Christmas level was included in the models).

All analyses were performed in Stata 14 [46], except the time series models that were fitted in R [47]. In line with current statistical thinking [48] and due to pilot nature of this study, we base our interpretations on effect sizes, and their precision, rather than emphasising the statistical significance relative to any boundary. We also provide *P* values for transparency.

## 3. Results

### 3.1 Participant characteristics

A total of 150 women aged 18 to 45 years who regularly shopped at 1 of the 6 study stores were recruited; 121 participants reported living with children (aged <18 years), and 72 of these participants also provided data about their child aged 2 to 6 years. The most successful recruitment methods were posted letters (49%) and in-store recruitment (34%). Attrition rates were low: 8% at 3-month and 14% at 6-month follow-ups. There were no significant differences in participant characteristics at baseline between intervention and control participants (Table 1). Participants’ median age was 36 years, 91% were white British, 59% had low educational attainment (no qualifications beyond age 16 years), and 46% were in paid employment. Almost half reported that the collaborating supermarket chain was where they purchased most of their groceries (44%).

**Table 1:** Baseline characteristics of 150 participants.

Characteristic	Total (*n* = 150)	Control (*n* = 88)	Intervention (*n* = 62)	*P* value
Age (years), median (IQR)	36.2 (31.4, 41.1)	36.1 (31.4, 40.9)	36.4 (31.3, 41.8)	0.85
White ethnicity, % (*n*)	91% (137)	91% (80)	92% (57)	0.83
Married, % (*n*)	43% (64)	42% (37)	44% (27)	0.56
Low education (no qualifications beyond age 16), % (*n*)	59% (87)	56% (49)	62% (38)	0.93
Most deprived half of neighbourhood deprivation (IMD), % (*n*)	79% (118)	82% (72)	74% (46)	0.26
Paid employment, % (*n*)	46% (68)	49% (43)	41% (25)	0.34
Most groceries from collaborating chain supermarket, % (*n*)	44% (66)	44% (39)	44% (27)	0.93
Pounds (£) spent on food per week, median (IQR)	75 (50, 100)	73 (50, 98)	80 (50, 100)	0.23
Provided data about child aged 2–6 years, % (*n*)	44% (66)	43% (38)	45% (28)	0.81

IMD, Index of Multiple Deprivation; IQR, interquartile range.

### 3.2 Store sales

**Fig 1:**
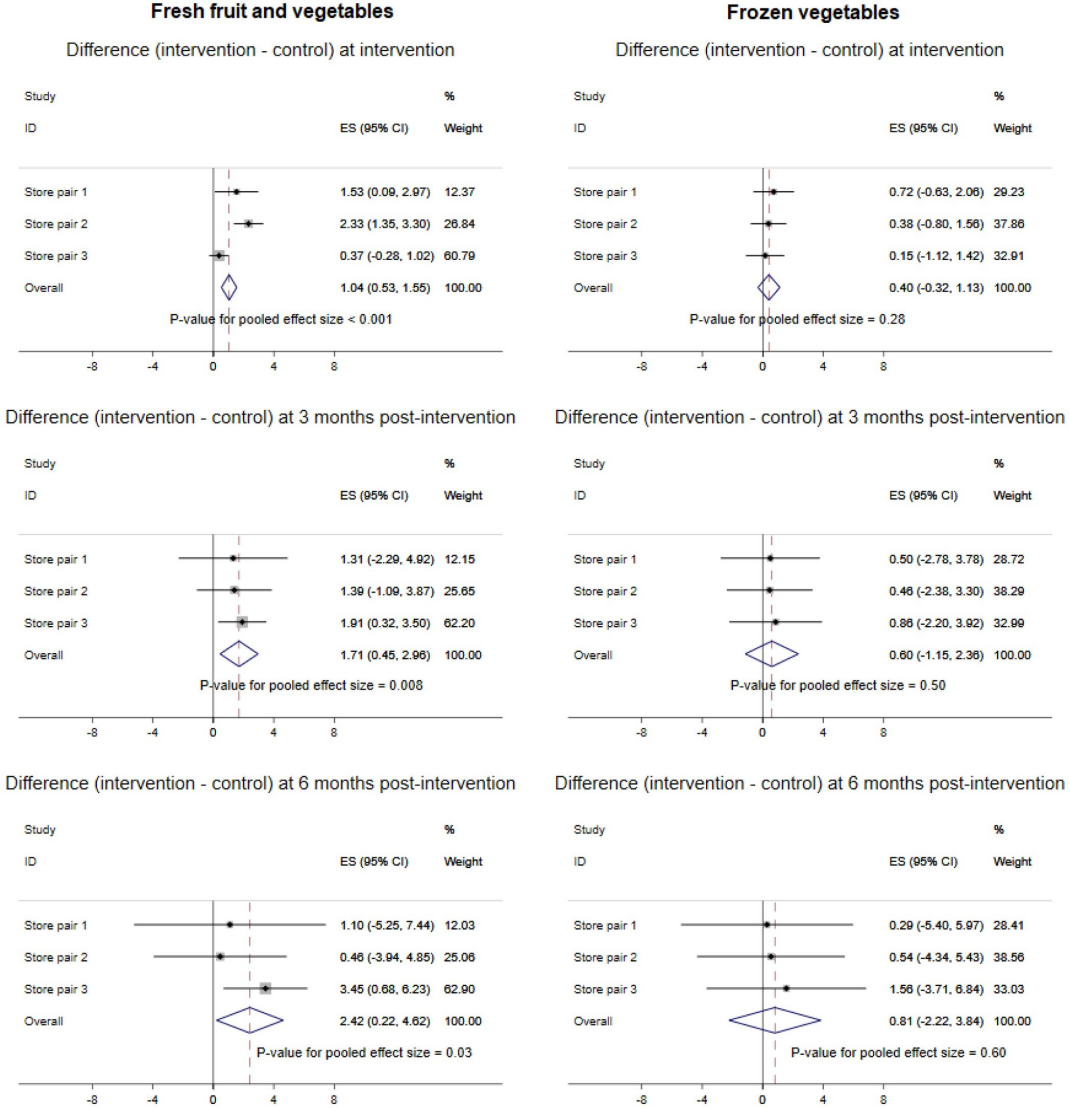
Meta-analysis of total sales of fresh fruits and vegetables and frozen vegetables by store status.

**Fig 2:**
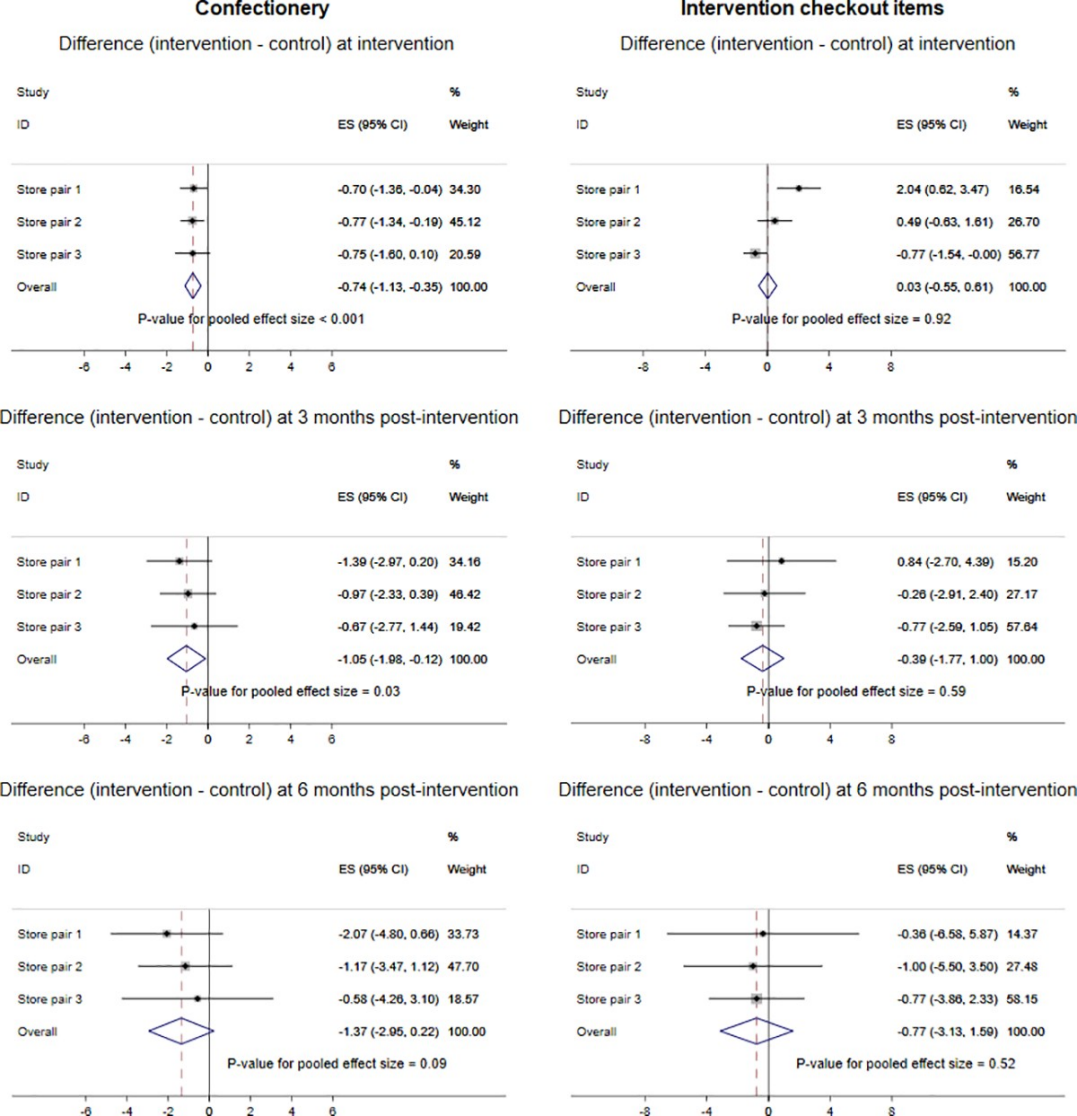
Meta-analysis of total sales of confectionery and intervention checkout items by store status.

The average number of items sold per week per store (in SDs) in store pairs is shown in Figs D and E in S1 File (complete data from all 6 stores); the synthesised results using meta-analysis are shown in Figs 1 and 2. Increases in sales of fresh fruits and vegetables were significantly greater in the intervention stores than would be predicted by the model counterfactuals at the time of intervention (difference = 1.04 SDs (95% CI 0.53, 1.55), *P* < 0.001), 3 months postintervention (difference = 1.71 SDs (95% CI 0.45, 2.96), *P* = 0.01), and 6 months postintervention (difference = 2.42 SDs (95% CI 0.22, 4.62), *P* = 0.03). These changes are approximately equivalent to 6,170 and 9,820 extra fruit and vegetable portions per store, per week at 3 and 6 months, respectively. Sales of frozen vegetables showed only very small increases at each time point. There were greater decreases in sales of confectionery in the intervention stores than would be predicted by the model counterfactuals at the time of intervention (difference = −0.74 SDs (95% CI −1.13, −0.35), *P* < 0.001), at 3 months postintervention (difference = −1.05 SDs (95% CI −1.98, −0.12), *P* = 0.03), and at 6 months postintervention (difference = −1.37 SDs (95% CI −2.95, 0.22), *P* = 0.09). These changes are approximately equivalent to 1,359 and 1,575 fewer confectionery portions per store, per week at 3 and 6 months, respectively. Sales of intervention checkout items showed inconsistent differences with little change at the time of the intervention (difference = 0.03 SDs (95% CI −0.55, 0.61), *P* = 0.92) and decreases at the 2 postintervention time points (difference at 3 months = −0.39 SDs (95% CI −1.77, 1.00), *P* = 0.59; difference at 6 months = −0.77 SDs (95% CI −3.13, 1.59), *P* = 0.52). The ICC of fruit and vegetable sales was 0.78 (95% CI 0.52, 0.93).

### 3.3 Individual purchasing

Of the 150 participants, 107 women (1,539 visits) had individual purchasing data from their loyalty cards for the study period, 56 recruited from control stores (851 visits) and 51 from intervention stores (688 visits). These missing data relate to the need for explicit consent from participants to the supermarket for them to legally share their loyalty card data; not all participants completed this additional consenting step. Of the 1,539 visits, 253 (140 control women visits and 113 intervention women visits) were not at the stores the women were recruited from. All 253 visits to alternative stores were to nonstudy stores, meaning that at 113 visits, the intervention women were not exposed to the store changes.

**Fig 3:**
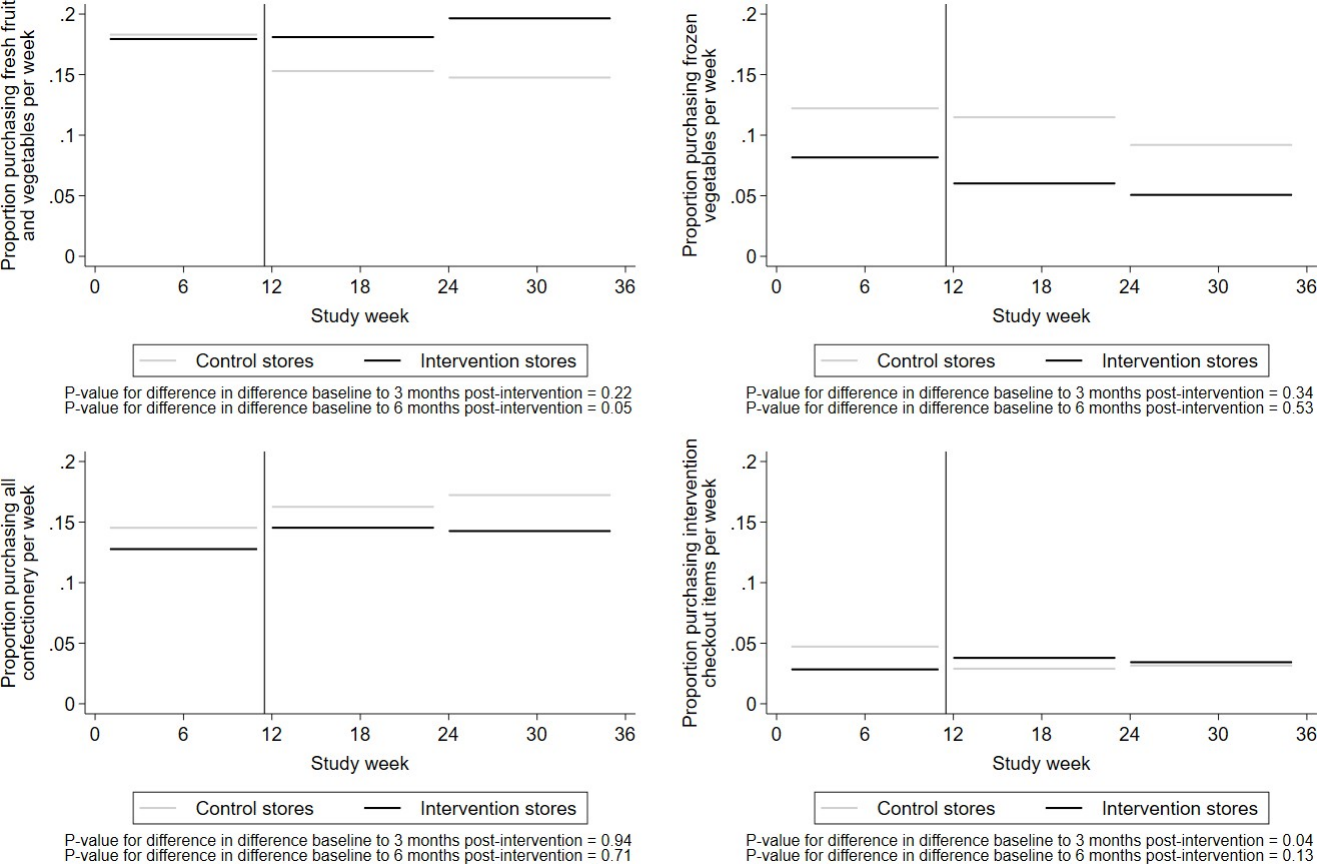
Modelled proportion of women purchasing food items in intervention and control stores.

Modelled proportions of women purchasing food items are shown in Fig 3. The proportion of purchasing fresh fruits and vegetables per week rose in intervention stores 3 months postintervention (0.2% (95% CI −3.6%, 3.9%)) compared to a drop in control stores (−3.0% (−6.6%, 0.6%)) (*P* = 0.22); even greater changes were seen 6 months postintervention (1.7% (−2.2%, 5.6%) in intervention stores compared to −3.5% (−7.2%, 0.1%) in control stores, *P* = 0.05). Frozen vegetable purchasing patterns demonstrated a decrease from baseline to 3 months postintervention among women in both intervention (−2.1% (95% CI −4.9%, 0.6%)) and control groups (−0.1% (95% CI −3.9%, 0.2%)) (*P* = 0.34) and from baseline to 6 months postintervention among women in both intervention (−3.0% (95% CI −6.1%, 0.1%)) and control groups (−3.1% (95% CI −5.9%, 0.3%)) (*P* = 0.53). Purchasing patterns for confectionery was also similar among women in intervention and control groups showing an increase from baseline to 3 months (*P* = 0.94) and 6 months (*P* = 0.71) postintervention. The proportion of women purchasing intervention checkout items were much lower in the intervention than control stores at baseline, but rose among intervention women by 1.0% (95% CI −0.9%, 2.8%) and 0.6% (−1.3%, 2.4%) in the 3 and 6 months postintervention periods, respectively, compared to a fall of −1.8% (−3.8%, 0.2%) and −1.6% (−3.6%, 0.5%) among control women (*P* = 0.04 and *P* = 0.13), respectively.

### 3.4 Dietary variables

Differences in women’s and children’s diets over time between groups are presented in Table 2. After adjustment for IMD, diet quality score at 3 months follow-up increased from baseline by 0.29 SDs ((95% CI 0.01, 0.57), *P* = 0.04) among intervention women compared to those shopping in control stores. This difference is equivalent to approximately 6 additional portions of green salad vegetables per week. After adjustment for all confounders, the size of this effect was only slightly attenuated (β = 0.26 (−0.01, 0.54), *P* = 0.06). A similar, although smaller, trend was observed among women at 6-month follow-up (β = 0.10 (−0.17, 0.37), *P* = 0.48) and among children, with the diet quality of intervention children increasing from baseline to 3 months (β = 0.15 (−0.28, 0.59), *P* = 0.49) and 6 months (β = 0.07 (−0.32, 0.46), *P* = 0.71) follow-up compared to control children. Women’s reported daily intake of fruits and vegetables increased, among intervention compared to control women, from baseline to 3 and 6 months follow-up, with greater increase observed at the later follow-up. Reported weekly confectionery intake decreased from baseline to 3 months follow-up among intervention women, compared to control women, but increased from baseline to 6 months follow-up.

**Table 2:** Effect of intervention on women’s and children’s dietary change from baseline to 3- and 6-month follow-up postintervention.

	Unadjusted*				Adjusted^†^			
Outcome	Beta	(95% CI)	*P* value	*n*	Beta	(95% CI)	*P* value	*n*
**Baseline to 3-month follow-up**								
Women’s diet quality score (SDs)	0.29	(0.01, 0.57)	0.04	138	0.26	(−0.01, 0.54)	0.06	132
Children’s diet quality score (SDs)	0.19	(−0.24, 0.62)	0.39	65	0.15	(−0.28, 0.59)	0.49	63
Women’s fruits and vegetables (portions per day)	0.31	(−0.40, 1.02)	0.39	136	0.14	(−0.56, 0.85)	0.69	130
Women’s confectionery (times per week)	−0.34	(−1.04, 0.35)	0.33	136	−0.27	(−0.98, 0.45)	0.46	130
**Baseline to 6-month follow-up**								
Women’s diet quality score (SDs)	0.10	(−0.16, 0.36)	0.45	125	0.10	(−0.17, 0.37)	0.48	119
Children’s diet quality score (SDs)	0.13	(−0.27, 0.52)	0.52	55	0.07	(−0.32, 0.46)	0.71	53
Women’s fruits and vegetables (portions per day)	0.64	(−0.21, 1.50)	0.14	128	0.50	(−0.33, 1.34)	0.23	122
Women’s confectionery (times per week)	0.32	(−0.56, 1.19)	0.47	127	0.29	(−0.62, 1.20)	0.54	121

* Unadjusted = adjusted for IMD (to control for similarities between pairs of stores).

^†^ Adjusted for IMD, age, money spent on groceries, children in household, and woman’s education.

IMD, Index of Multiple Deprivation; SD, standard deviation.

### 3.5 Economic impacts

There was a relatively small difference in the mean (SD) change from baseline to 3- or 6-month follow-ups in the amount of money women reported spending on grocery foods in the intervention group (−£7.63 (£26.08) and −£1.73 (£27.97), respectively) compared to the control group (−£3.93 (£22.36) and £3.62 (£41.58), respectively) (*P* = 0.80 and *P* = 0.91, respectively). The intervention also made little difference to women’s total weekly spend as recorded by loyalty cards; the mean (SD) changes from baseline to 3- and 6-month follow-ups in the intervention group were −£0.08 (£7.09) and −£1.26 (£9.43), respectively, compared to −£0.66 (£7.25) and −£2.34 (£7.83) in the control group (*P* = 0.89 and *P* = 0.77), respectively.

There was little impact of the intervention on total weekly store sales, with overall differences in stores sales between intervention and control stores of −0.01 SDs (95% CI −1.22, 1.21), *P* = 0.99 at the time of the intervention, −0.04 SDs (−1.21, 1.14), *P* = 0.95 at 3 months postintervention and 0.21 SDs (−1.79, 2.21), *P* = 0.84 at 6 months postintervention.

### 3.6 Fidelity assessment

The fruit and vegetable components of the intervention were fully implemented in all intervention stores. Stock levels of nonfood items at checkouts were reported and observed to be lower than anticipated in 2 of the intervention stores during the 3-6-month postintervention period. These issues were attributed, at least in part, to demand outweighing supply of the designated intervention checkout items.

## 4. Discussion

### 4.1 Principal findings

This pilot supermarket trial showed that creating a healthier store layout by expanding the range of fruits and vegetables and placing them near the entrance, plus removing all unhealthy foods, namely confectionery, from checkouts and aisle ends opposite checkouts had a positive effect for health benefit, increasing fresh fruit and vegetable sales and reducing confectionery sales at a population (store) level. Among a sample of women customers of childbearing age, the intervention showed beneficial effects on fresh fruit and vegetable purchasing patterns, particularly after 6 months of intervention implementation, but no intervention effect was observed for participants’ confectionery purchases. Nonfood items, water, and sugar-free gum, which were placed at checkouts during the intervention, were purchased more by trial participants exposed to the intervention, but did not translate into increased sales of these items at the store level. Findings from assessment of the impact of the intervention on women’s and children’s diets were more equivocal. Trends for the dietary variables were predominantly in the expected direction for health benefit, with improvements in women’s overall dietary quality and daily fruit and vegetable intake at 3 months follow-up, particularly noteworthy for size of dietary change. The economic analysis showed virtually no impact on weekly household grocery spend across all participants or overall weekly store sales across all stores, indicating no detrimental cost effect of the intervention to participants or the retailer.

### 4.2 Strengths and limitations

To our knowledge, this is the first health-focused product placement study in supermarkets to use (i) loyalty card data to track effects on household purchasing patterns of existing customers in addition to store sales data to measure population-level effects; and (ii) dietary data from more than 1 household member to aid understanding of who has been affected by the intervention. This study has a number of other advantages over existing supermarket placement research [13] including use of a matched comparison group; use of robust statistical analysis methods, particularly the interrupted time series approach to assess intervention effects on store sales; and the study setting and sample including a high representation of families from lower socioeconomic backgrounds, thus providing important information on interventions that have potential to reduce inequalities. The intervention included both reduced availability and prominence of unhealthy foods and improved availability and prominence of fruits and vegetables, plus it trialled the use of nonfood items at checkouts in an effort to maintain business profitability while reducing customers’ calorie purchasing opportunities.

This study has a number of limitations. It was not possible to randomise stores to intervention or control groups; thus, the study may be biased by unmeasured confounding effects. Parallel designs, like the one used in our study, with control groups matched on area characteristics and store sales (plus adjustment for confounders), however, do offer a robust design in real-world settings and enables valuable knowledge of intervention effectiveness in complex social contexts to be shared with policy makers, particularly with data collected at store, household, and individual levels. Although the number of stores and participants that took part in this study is consistent with previous placement intervention research [17,18,24], this was a pilot study and is underpowered because store-based placement intervention studies require power calculations that take account of clustering at the store level, so the study results should be viewed with some caution. Nevertheless, the study’s effect sizes demonstrated meaningful improvements in the healthiness of store sales and in household purchases of fresh produce.

Store selection and intervention implementation were not within the control of the research team, and some issues were identified. Under- or overestimation of intervention effects observed may therefore be possible. Finally, the economic analysis was limited in scope and did not include broader cost implications such as time or travel costs for individuals or profit loss, infrastructure, and staff training costs for the retailer, nor were the benefits of improved dietary quality on health or well-being calculated.

### 4.3 Comparison with previous literature

Previous intervention studies that repositioned fresh fruits and vegetables to prominent locations in food stores have found effects in a similar direction, but smaller in magnitude than the potentially meaningful effect sizes observed in our study [23,24,49]. One study set in discount supermarkets in Denmark failed to demonstrate significant intervention effects on store sales. This finding may relate to the intervention involving prominent positioning of additional produce bin displays rather than repositioning of the entire produce section near the store entrance and the shorter 3-month intervention duration.

Our study showed stronger intervention effects at 6 months than at 3 months follow-up, suggesting that changes in food shopping, like other health behaviours, take approximately 14 weeks for habit formation and strengthen over time [50]. Another study, in convenience stores in the US, which moved the fresh produce section to intervention stores’ entrances, found that fruit and vegetable sales from government assistance vouchers for young, low-income families increased in intervention stores during the 5-month intervention, yet decreased in control stores (difference of US$63/month between groups) [24]. Women’s fresh fruit and vegetable purchasing patterns from loyalty card transactions in our study similarly revealed increasing and decreasing trends for intervention and control participants, respectively; placing the produce section in a prominent in-store position may have protective benefits as indicated by the improvements in intervention women’s dietary quality. This finding could be of particular importance because long-term analyses of UK purchases of fresh fruits and vegetables show a general decline: Fresh green vegetable purchases dropped 25% between 2005 and 2015, and fresh fruit purchases decreased by 17% from 2006 to 2015 [51].

Our finding, that the intervention had a beneficial effect at reducing confectionery sales at the store level, differs in strength of effect from previous checkout intervention research from the Netherlands [16], US [17], Denmark [18], and Norway [52]. This difference may be attributable to the fact that previous interventions positioned healthier products either alongside or at alternative checkouts to existing unhealthy foods or beverages, rather than removing them completely. Additionally, our study also removed confectionery from the aisle ends opposite checkouts. This extra intervention component has not been tested previously and may have helped to enhance store-level intervention effects because more than two-thirds of products positioned in this location in UK supermarkets are unhealthy foods and beverages [53].

Despite the healthier design of our intervention compared to others, it did not influence the confectionery purchasing and intake patterns of women participants. Intense marketing of confectionery products occurs throughout supermarkets, not just in the checkout area, and it is probable that our study’s participants were tempted by the additional positioning of confectionery products at the store entrance and freestanding aisle displays [12]. National household purchasing trends reveal that confectionery spending continues to increase, rising 10% from 2012 to 2015 and a further 6% from 2016 to 2018 [54]. Many national celebrations have become symbolised by confectionery. During the course of our study’s follow-up period, our participants celebrated Halloween, Christmas, Valentine’s Day, and Mother’s Day. While Easter did not fall within our study period, Easter-related treats are on sale from early January and would have been available for our participants to purchase. Research commissioned by the Royal Society for Public Health found strong customer opinions against sustained marketing of seasonal confectionery, with two-thirds of people agreeing that special occasions are used too much to advertise and sell unhealthy food and one-third stating that this strategy prompts them to have poorer diets than they normally would [55].

The inclusion of frozen vegetables as part of the healthier store layout intervention was novel, and, although our results did not indicate a beneficial effect on store sales or customer purchasing, no detrimental substitution effects were observed. Prominent positioning of lower calorie frozen ready meals has shown to increase sales of these products [17], suggesting that future research could further test the effect of enhancing placement of frozen vegetables. Such studies may be particularly pertinent when food system shocks, such as no-deal Brexit or COVID-19 seasonal agricultural workforce concerns, have the potential to hinder fresh produce availability [56].

### 4.4 Implications for policy

In response to COVID-19, international experts are calling for strong government leadership to recalibrate economic activities in the food system for public health and the public good [57]. A positive example of political action includes the British Prime Minister, Boris Johnson, declaring he now supports a more interventionist approach to tackle obesity in the wake of his experience of contracting COVID-19, attributing his need for intensive care to be, at least partly, due to his being overweight [58]. Our findings can thus provide a national, contextually relevant contribution to public policy, tentatively indicating that the planned ban on the use of prominent placement strategies for unhealthy food and drink products in supermarkets and other outlets could reduce sales of unhealthy foods like confectionery at a population level. Moreover, our findings suggest that expanding the regulation to include the placement of the produce section near store entrances in all supermarkets could increase fruit and vegetable sales, including among more disadvantaged families, and further enhance the potential benefits of the regulation to population diet. The regulation may be even more effective at limiting impulsive calorie sales if only nonfood products were to be sold in certain prominent in-store locations. Our study findings suggest that nonfood products are purchased more by customers when positioned at checkouts in place of confectionery, but adequate variety and sustained supply of nonfood products needs careful consideration to ensure viability for the retailer. This represents an important question for future research, alongside conducting a full economic evaluation from the individual, retailer, and society perspective.

### 4.5 Directions for future research

The body of evidence would benefit if future intervention research studies could be adequately powered; such studies are rare and require considerable commitment from retailers [59]. Novel trial designs using routinely collected data (e.g., loyalty card transactions within the boundaries of data protection regulations) or longitudinal observational studies may be appropriate alternatives to measure policy directives or other natural experiments [60]. Future research might also consider assessing the independent and additive effects of altering products at different prominent in-store locations and the effects among different population groups to enhance understanding of the most effective strategies and for whom. Qualitative research with category managers who make decisions about prominent product placement could help identify existing food system drivers for unhealthy foods and opportunities/plans for changing to nonfood or healthier products, particularly in the context of looming government legislation.

## 5. Conclusions

Although a pilot study, these results provide a comprehensive assessment of an intervention to create a healthier store layout in discount supermarkets by presenting effects on store sales, customer loyalty card purchasing patterns, and the diets of more than 1 household member. The results showed a reduction in confectionery, and increase in fresh fruits and vegetables, store sales when nonfood items and water were placed at all checkouts and aisle ends opposite, and an enhanced produce section was repositioned to near the store entrance. Beneficial effects were also observed for household fruit and vegetable purchasing and women’s dietary quality. This study therefore provides novel evidence to suggest that the intended UK government ban on prominent placement of unhealthy foods across retail outlets could be beneficial for population diet and that effects may be further enhanced if requirements for a produce section near supermarket entrances were incorporated into the regulation.

## Supporting information

S1 File Supporting information figures. Fig A: Flow diagram. Fig B: DAG for the causal relationship between intervention and women’s dietary change. Fig C: DAG for the causal relationship between intervention and child’s dietary change. Fig D: Interrupted time series for store pairs of total sales of fresh fruits and vegetables and frozen vegetables. Fig E: Interrupted time series for store pairs of total sales of confectionery and intervention checkout items by store status. DAG, directed acyclic graph.
(DOCX)

## Abbreviations

COVID-19: Coronavirus Disease 2019
DAG: directed acyclic graph
FFQ: Food Frequency Questionnaire
ICC: intraclass correlation coefficient
IMD: Index of Multiple Deprivation
IQR: interquartile range
SD: standard deviation
