# Supplementary material for: Altering product placement to create a healthier layout in supermarkets: Outcomes on store sales, customer purchasing, and diet in a prospective matched controlled cluster study
Source: PLoS Med. 2021 Sep 7;18(9):e1003729. doi: 10.1371/journal.pmed.1003729 (PMC8423266; doi:10.1371/journal.pmed.1003729)
Supplement: S1 File — Fig A: Flow diagram. Fig B: DAG for the causal relationship between intervention and women’s dietary change. Fig C: DAG for the causal relationship between intervention and child’s dietary change. Fig D: Interrupted time series for store pairs of total sales of fresh fruits and vegetables and frozen vegetables. Fig E: Interrupted time series for store pairs of total sales of confectionery and intervention checkout items by store status. DAG, directed acyclic graph. (DOCX) [file pmed.1003729.s001.docx]

**S1 Fig A Flow diagram**

Excluded (n=54)

-not met criteria (n=11)

-declined/other (n=43)

Lost to follow-up (n=6)

Lost to follow-up (n=4)

Lost to follow-up (n=5)

Lost to follow-up (n=6)

**6 month follow-up and analysis**

Purchasing data (n=56)

Dietary data (n=51)

Purchasing data (n=51)

Dietary data (n=78)

Purchasing data (n=56)

Dietary data (n=56)

Purchasing data (n=51)

Dietary data (n=82)

**3 month follow-up and analysis**

Dietary data (n=62)

Purchasing data (n=51)

Dietary data (n=88)

Purchasing data (n=56)

**Baseline assessment** n=150

**Women assessed for eligibility**

n=204

**Intervention**

**n=3 stores**

**Control**

**n=3 stores**

Intervention stores from refurbishment schedule

Control stores matched to an intervention store (area deprivation, sales & customer profile)

**Recruitment of stores**

n= 6

**S1 Fig B Directed Acyclic Graph for the causal relationship between intervention and women’s dietary change**

**
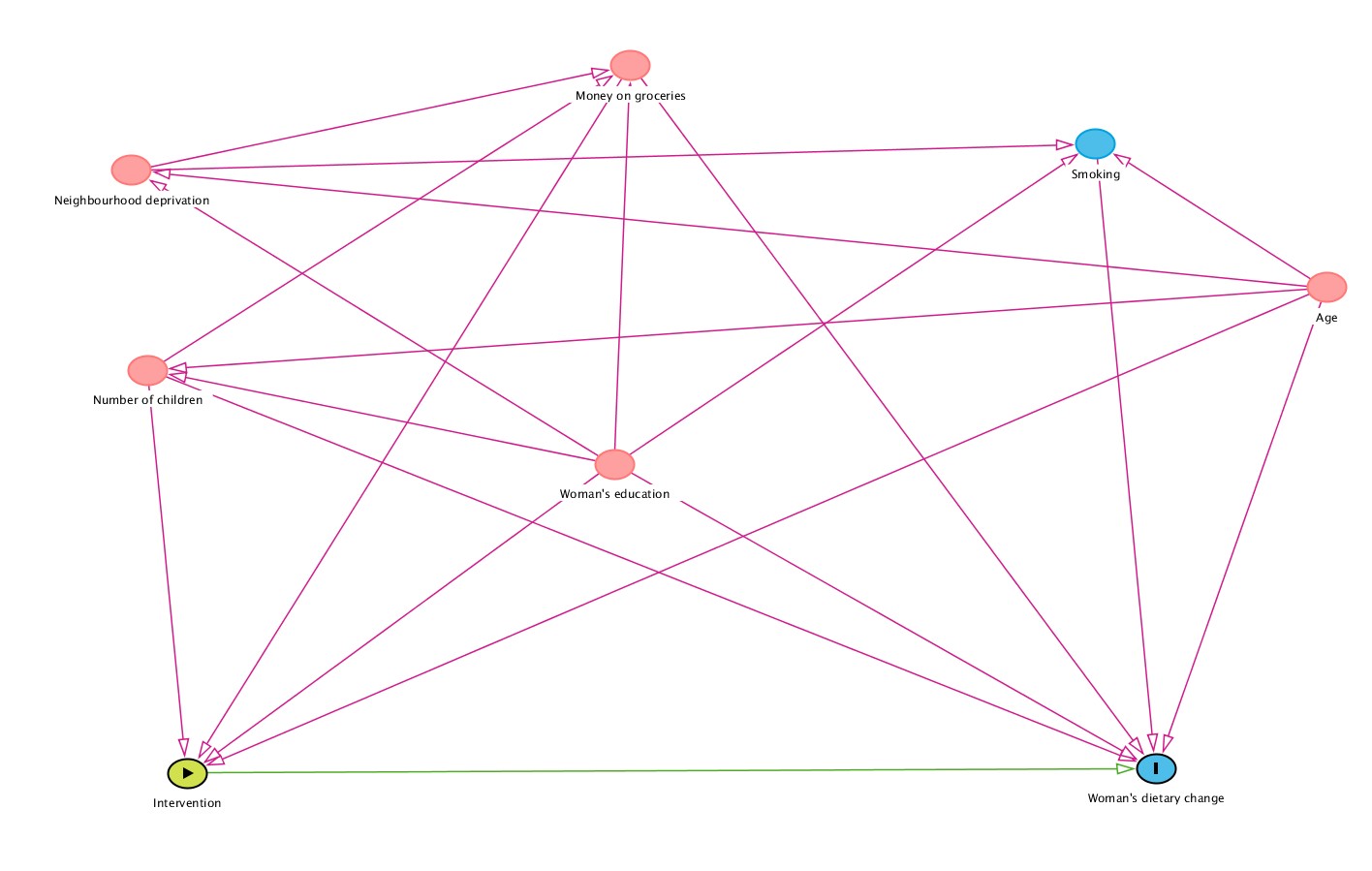
**

**
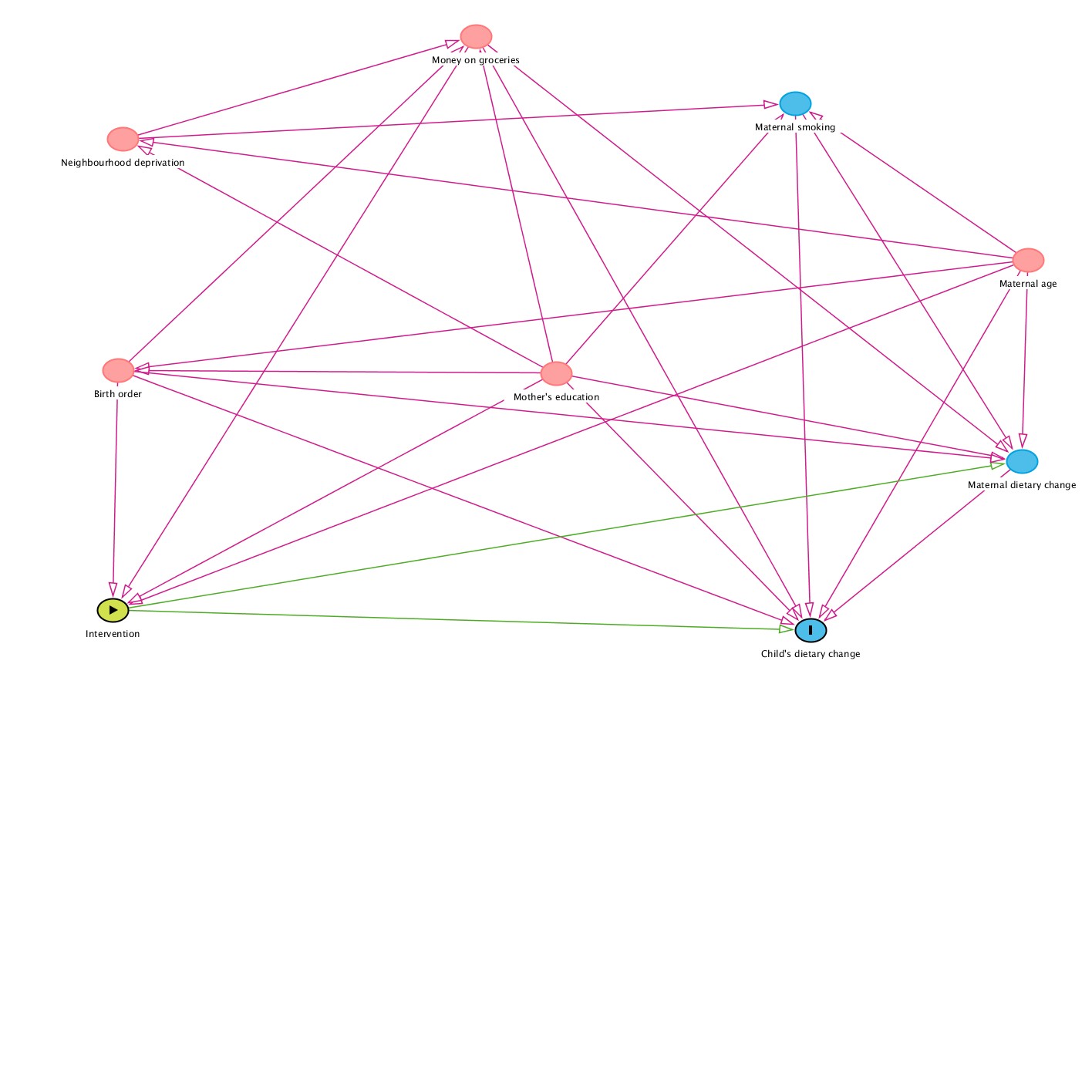
S1 Fig C Directed Acyclic Graph for the causal relationship between intervention and child’s dietary change**

**S1 Fig D** **Interrupted time series for store pairs** **of total sales of fresh fruit and vegetables and frozen vegetables**

**S1 Fig E Interrupted time series for store pairs of total sales of confectionery and intervention checkout items by store status**
